# Insects of French Guiana: a baseline for diversity and taxonomic effort

**DOI:** 10.3897/zookeys.434.7582

**Published:** 2014-08-14

**Authors:** Stéphane Brûlé, Julien Touroult

**Affiliations:** 1 Société entomologique Antilles-Guyane 69, Allée du Bois, 38890 Salagnon, France; 2 Service du Patrimoine Naturel, Muséum national d'Histoire naturelle, CP41, 36 rue Geoffroy Saint-Hilaire, 75 005 Paris, France

**Keywords:** Regional checklist, Neotropics, species database, Guiana Shield, biodiversity, arthropods, taxonomic diversity, endemism, taxonomic effort, Linnean shortfall

## Abstract

This paper analyzes the first checklist of insects from French Guiana. Compiled by a group of 70 experts based on published records, it comprises about 15 100 valid species names belonging to 20 orders and 322 families. Currently, about 17% of the species are only known from French Guiana or from the Guiana Shield region. Since Linné, the average rate of description has been 59 species per year, which has been increasing in the last 10 years. Based on a sample of recent taxonomic and faunistic papers covering 736 new species for French Guiana, 46% of the species came from new country records, the rest from new species descriptions. The rate of faunistic progress (new species or new records) is about 180 species per year over the last five years. Sixty-five percent of these faunal records came from non-professional entomologists and 74% of the holotypes of new species were collected by amateur entomologists. A rough extrapolation, using two different methods, provides a likely estimate of around 100 000 species, the most conservative estimate being 67 000 species and the highest 184 000. Therefore, an estimated 80% of the species remain to be recorded and, in a best-case scenario, at least 270 years would be needed to complete the biotic inventory, at the current rate of species descriptions and distribution records. Although no order is exhaustively inventoried, the most in need of study are Diptera, Hymenoptera, Hemiptera and Trichoptera; and in absolute numbers, Coleoptera. These results and the fundamental role of non-professional entomologists in collecting and describing new species are discussed.

## Introduction

The total number of arthropod species in tropical forests has been the object of intense speculation and scientific debate since the extrapolation published by Erwin (1982). Although knowing how many species are present is not required for research and applied conservation of biodiversity (Magurran and Queiroz 2010), it is still a great intellectual challenge to evaluate the actual diversity of life on earth.

French Guiana is a small territory of 84 000 km^2^ in northern South America. It is in the Guianan moist forest ecoregion (Dinerstein et al. 1995), and is 95% covered by relatively homogeneous lowland tropical rain forest (Guitet et al. 2013). A recent book dealing with French Guiana’s biodiversity (Charles-Dominique 2011) indicates that 100 000 species of insects are known so far, with a total number probably between 400 000 and 1 million species. This estimation, without any published references, seems quite unlikely to entomologists studying the French Guianan fauna. However, it highlighted the fact that no global checklist was available to provide an accurate account of what was known so far. French Guiana, with its rich diversity, became a popular destination for entomologists worldwide and therefore is assumed to be better studied than some other tropical countries. Unfortunately, faunal lists are only available for a restricted number of groups (e.g. Coleoptera: Cerambycidae, Tavakilian and Chevillotte 2012).

There are at least three important objectives for this work on the compilation of regional species databases:

1) To enable data exchange, which makes data sharing possible between observational or collection databases from different users, as long as they use the same taxonomic list. This data sharing is the basis for filling the “Wallacean shortfall”, which aims to study distribution, biogeographic questions and applied questions of conservation (Cardoso et al. 2011), such as defining priority areas in systematic conservation planning (Margules et al. 2002).

2) To have global indicators of diversity and to monitor taxonomic progress, which facilitates communication with the general public and the biodiversity policy makers about invertebrate biodiversity and the challenge of the “Linnean shortfall”, that is to say that only fraction of the planet's species has been described by science (Whittaker et al. 2005).

3) To facilitate the work of taxonomists, by enabling them to easily check what is already known and to stimulate further taxonomic research and the publication of new country records.

For these reasons, we launched this project as a part of the national taxonomic database (TAXREF: Gargominy et al. 2013) on behalf of the natural heritage inventory of France (MNHN, online). The database will be available online (see Suppl. material 1) and will be updated on a yearly basis. This publication provides descriptive analysis of what is known so far, quantifies the description rates, discusses who supports the faunistic and taxonomic progress, which are the most and least studied orders, and tries to extrapolate a rough figure of the total species richness of insects in French Guiana.

## Material and methods

### Elaboration of the checklist of known species

Except for a few families that recently benefited from a regional checklist (e.g. Heiss and Moragues 2009, Faynel 2010, Brûlé 2011, Pauly et al. 2013) and some global species databases (e.g. Tavakilian and Chevillotte 2012), no list of species was available for French Guiana. In 2008, we started the French Guiana list, as a part of the French national taxonomic database (TAXREF: Gargominy et al. 2013), with the help of all our colleagues working on specimens collected by SEAG (Société entomologique Antilles-Guyane — Entomological Society of French Guiana and West Indies). At the end of 2012, we asked those experts to compile and transmit lists in their taxonomic groups of study, taking into account only published records for French Guiana up to the beginning of 2013. Eighty experts, listed in the acknowledgements section, took up the challenge and provided updated lists for 169 families, representing 53% of the total families and 90% of the total species in the final list. The other orders and families, not listed by a specialist but suspected to be present in French Guiana, were compiled by the authors. This was done by searching for relevant references on Google Scholar and the Zoological Record. It is likely that, for those groups, certain lists are incomplete and some species names are obsolete.

The lists were compiled in a spreadsheet with the taxonomic hierarchy of family, subfamily, tribe (occasionally), genus, species, subspecies (occasionally), and author's name for each taxon. For this first effort, we focused only on valid taxa. These lists were coded using the TAXREF format (Gargominy et al. 2013) in order to integrate the species database. This includes a “biogeographic status” field regarding the occurrence of the species in French Guiana. The following codes were used: P: taxa present in French Guiana (default status); G: taxa described and only known (so far) from French Guiana; E: endemic taxa (used only for well-known groups, otherwise G); S: sub-endemic taxa (well-known taxa ranging only in the Guiana Shield region); I: introduced taxa; J: introduced and invasive; B: vagrant; Q: taxa recorded from French Guiana in error.

### Other insect baselines for comparison

To compare and extrapolate the total diversity of French Guiana, we first sought published checklists or available online databases for very well-known fauna, in all orders and families, in as close a biogeographical context (tropical America) as possible. We found no convincing resources corresponding to these criteria. We finally used for comparison three faunal references: total species richness of the global, Nearctic and French faunas.

The total number of known species per order worldwide (Zhang 2013) cannot be used to extrapolate total diversity, as it is dependent on the level of the taxonomic knowledge. However, it can be used to identify major gaps, by comparing the number of species known worldwide per order with the same figure from French Guiana.

The figures for the North American fauna from the Nomina Insecta Nearctica series (Poole and Gentili 1996) were used at the family level. Although compiled 15 years ago, this series provides a comprehensive reference for all orders. Knowing the total figure for North America (ca. 95 000 species), it was possible to estimate the diversity in French Guiana using cross-multiplication from a sample of well-known families. These figures are useful, but we assume that the total richness based on this projection is surely underestimated.

The Fauna Europeae project is a continent-wide data basing effort (De Jong 2013). Additional faunistic and taxonomic updates have been conducted on a subset of the TAXREF data limited to France and Corsica. The French fauna is one of the longest studied, and estimated to contain 36 000 insect species. This total can be considered accurate, however new species are added every year, especially restricted range species (Essl et al. 2013).

### Extrapolating a rough estimate of total diversity

We use a method similar to the one use by Lewinsohn and Prado (2005) for Brazil, which consists of comparing some groups with a benchmark fauna and estimating the total using a rule of proportionality. Instead of using as a benchmark the extrapolated worldwide diversity, we use two well-known temperate faunas, one of the possible methods reviewed by Mora et al. (2011).

First, experts were asked to provide their best estimate of total number of species, by counting the number of unnamed « sp. » and by extrapolating the number of unseen species. Three estimates were calculated: the minimum, probable « best guess » and maximum number of species. The minimal number is a conservative figure, corresponding roughly to the number of different morphospecies seen from French Guiana by the expert, generally from a huge sample of localities and specimens. In a few cases (5 out of 68) the probable number extrapolates the unseen species from the number of singletons and doubletons as in Chao 1 estimator (Colwell and Coddington 1994). In most cases, this was performed by an educated guess corresponding to the rate of new taxa seen. We received answers for 68 taxonomic groups at the ordinal (Dermaptera, Ephemeroptera, Phasmatodea, Odonata), superfamily (Hymenoptera: Chalcidoidea; Hemiptera: Pentatomoidea) and mostly the family level (62 families or subfamilies). Most of them are included in the richest orders at the world level according to Zhang (2013). We consider these groups as representative “random” samples of the total insect taxa.

Secondly, we compiled figures of species richness for the same taxonomic groups in benchmark faunas of North America (based on Poole and Gentili 1996) and of France from TAXREF (Gargominy et al. 2013). A few taxa have a lower richness in French Guiana (e.g. Coleoptera, Lucanidae; Hymenoptera, Apidae), but most show an equal or much higher diversity (see Suppl. material 2). All these groups were included in the analysis to avoid any bias towards more, or less diversified groups in French Guiana.

Thirdly, considering that the proportion of these 68 groups studied was representative of the ratio of richness between French Guiana and the benchmark fauna, we used a simple rule of proportionality to obtain rough estimates of the total diversity of species in French Guiana. For instance, consider that 1 000 species is the estimated total number of species in 10 well-known taxonomic groups in French Guiana; 500 is the reported number of species in the benchmark fauna for those same 10 groups and 100 000 species is the overall number of insect species in the benchmark well-known fauna, then the total extrapolation for French Guiana would be 200 000 species [(1000/500) × 100 000].

As there is no perfect method to assess species richness (Mora et al. 2011), we also used a second method to assess robustness of the estimate. Using the relatively scale-independent correlation found between vascular plant richness and arthropod richness in Panama (Basset et al. 2012), the minimum ratio between arthropods and plants is 17:1 and 20:1 is the most likely. For plant richness, we used the commonly admitted number of 5 750 species of vascular plants (Delnatte and Meyer 2012).

### Assessing descriptive and faunistic work in recent years

Belonging to the large Guianan moist forest ecoregion, French Guiana shares a species pool with adjacent countries. Species cited from French Guiana can either be described from French Guiana or from another country and then published as a new record for French Guiana. These new country records may be important to assess the real progress of faunistic knowledge. Therefore, we investigate a large sample of 144 faunistic and taxonomic papers dealing with French Guiana, from 2008 to 2013. For each of these, we compute the following items: number of new species and new country records, professional status and nationality of authors and professional status of the collector of the holotype and of all the material cited from French Guiana. To assess how many species were described or cited by status of the authors, we divide the number of species treated by the number of authors (see Hołyński 2013) and then sum by categories of authors: professional taxonomist, non-professional and para-professional. By para-professional, we mean individuals affiliated with an institution or biologists whose job is not taxonomy. We had an “unknown” category, which we did not include as it contained just one author.

## Results and discussion

### Known taxa by order

Of the 29 orders of insects currently recognized, excluding fossils (Zhang 2013), 20 taxa appeared in the first checklist for French Guiana. The nine orders not represented are Mecoptera, Archaeognatha, Zygentoma, Embioptera, Grylloblattodea, Mantophasmatodea (the latter two usually grouped in Notoptera in recent classifications), Zoraptera, Phthiraptera, and Raphidioptera. These are all minor orders regarding their species diversity, even if Phthiraptera reaches more than 5 600 species (Zhang 2013). The absence of species in the list could be either a genuine absence of these orders, a lack of publication on the subject or a lack of specific research in the literature on these groups.

**Table 1.** Overall number of taxa known by order in French Guiana.

Order	Subspecies	Species	Genera	Tribe	Subfamily	Family	Total
Blattodea		251	116		24	8	**399**
Coleoptera	66	5759	1863	343	167	81	**8301**
Dermaptera		45	21	1	9	3	**79**
Diptera	2	577	142	17	19	32	**789**
Ephemeroptera		20	15		1	4	**40**
Hemiptera	7	859	451	74	78	46	**1515**
Hymenoptera	32	1338	295	67	58	39	**1829**
Lepidoptera	561	5507	1541	52	91	48	**7800**
Mantodea		93	40	15	14	6	**168**
Megaloptera		5	2			1	**8**
Neuroptera		25	15		1	4	**45**
Odonata	13	237	84			14	**348**
Orthoptera	18	341	216	59	37	12	**683**
Phasmatodea		50	25	11	8	5	**99**
Plecoptera		1	1			**1**	**3**
Psocoptera		20	12			9	**41**
Siphonaptera	6	12	7			3	**28**
Strepsiptera		1	1			1	**3**
Thysanoptera		6	6		4	3	**19**
Trichoptera		36	14	2	6	2	**60**
**Total**	**705**	**15183**	**4867**	**641**	**517**	**322**	**22257**

Out of 22 257 taxa listed, about 30% were above the species level. Overall, 15 183 species are inventoried from French Guiana. The compilation and expertise for listing these species may not be comprehensive but the most diverse families are the focus of this work so there should not be too many species missed. At most, we estimate there could be 18 000 published species records from French Guiana.

Only 705 taxa at the subspecies level were mentioned from French Guiana. Lepidoptera accounts for 80% of these taxa, and of these most are Rhopalocera. This is probably due to the focus of entomologists on this well-known suborder.

### Introduced species and endemism

The fauna of French Guiana is believed to hold few real endemics because, as pointed out for its flora (Granville et al. 1996), there are no strong geographical barriers between French Guiana and neighboring countries (Suriname and Brasilian state of Amapá). However, we found about 17% of insect species are known only from French Guiana or the Guiana shield (considered as subendemics). This is a conservative figure, as this status has not been reported accurately for some orders or families. We considered 1 033 rather well known species (6.8% of the total) as likely endemics of the Guiana Shield. Of recently described species, 10.1%, are so far known only from their country of description, but may be considered subendemics or more widespread species as chorological knowledge progresses. A good example of a likely true subendemic is the large and attractive dynastine beetle *Ceratophileurus lemoulti* Ohaus, 1911, known only from French Guiana and Surinam (Gillett et al. 2010). We should be very cautious with levels of endemism, even at the level of the Guiana Shield, as many insects previously known only from French Guiana are also present in the Amazonian part of Andean countries, exhibiting what one botanist called a “peri-amazonian” distribution (Granville 1992). As an illustration, for Lepidoptera, probably the best studied group, less than 1% of the species are considered endemic. However, recently discovered species may be cryptic species, species from poorly studied groups or a truly restricted range species, making it difficult to assess real endemism in a context of incomplete inventories for South American insects.

**Table 2.** Repartition of French Guiana species among different biogeographical categories. “Described from French Guiana” represents taxa known only (so far) from French Guiana.

Order	Dubious records	Occasional, vagrant	Endemic or sub-endemic	Described from French Guiana	Introduced (not invasive)	Introduced (invasive)	Other “presence”
Blattodea	1	0	43	28	0	3	176
Coleoptera	78		812	844	5	5	4021
Dermaptera			3				42
Diptera	2		6	50			519
Ephemeroptera							20
Hemiptera			30	66			763
Hymenoptera	3		72	132			1131
Lepidoptera	11	1	49	265	3		5178
Mantodea			4	10			79
Megaloptera							5
Neuroptera							25
Odonata	2	1	14	2			218
Orthoptera				142			199
Phasmida							50
Plecoptera							1
Psocoptera							20
Siphonaptera							12
Strepsiptera							1
Thysanoptera							6
Trichoptera							36
**Total**	97	2	1033	1539	8	8	12502

The number of species reported as introduced in French Guiana is very low (16 species, about 0.1%), especially compared with checklists from the West Indies, which categorize about 5% of taxa as introduced (e.g. Peck 2011). This may come from a reporting bias, with either a lack of publication on agricultural pests in French Guiana or a lack of literature searching for existing papers. It is also probably genuine, due to the high integrity of the forest cover of French Guiana, which is generally not favorable for alien species establishment (Hooper et al. 2005, Delnatte and Meyer 2012).

### Rate of description

The rate of species descriptions is, on average, 59 valid taxa per year being added to the French Guiana fauna during the 255 years between Linné and today (2013). The highest peak was during the early twentieth century, with 178 species per year between 1904 and 1908. In the last century, the lowest period was between 1960 and 1970. In the last five years (2008-2012) the rate is nearly twice this average, with 102 species per year.

Description curves cannot be used for estimating total species richness (Bebber et al. 2007), but provide a good way to compare taxonomic effort between taxa (Fig. 2). The rate is quite different among orders. Currently, the rate of description is robust for Coleoptera, Hymenoptera and Hemiptera, and significantly higher than for Lepidoptera (Kolmogorov-Smirnov test for Lepidoptera vs Coleoptera: D = 0.19; p<0.001). The description rate seems to have nearly reached a figau for macro-Lepidoptera. The numerically smaller orders also exhibit a significantly different description rate pattern (Kolmogorov-Smirnov test for Hemiptera vs other orders: D = 0.22; p<0.001): the description effort was very low before 1900, quite intense between 1900 and 1960, and constant after 1960, nearly following the description rate for Coleoptera, Hemiptera and Hymenoptera.

The peak in the early 20^th^ century was due to the publication of books, mainly on Lepidoptera, which yielded numerous new species at the same time, whereas now, new species are typically described in journal articles, which results in many more papers and authors than before, but fewer species per paper (as pointed at the global level by Costello et al. 2013b).

**Figure 1.**
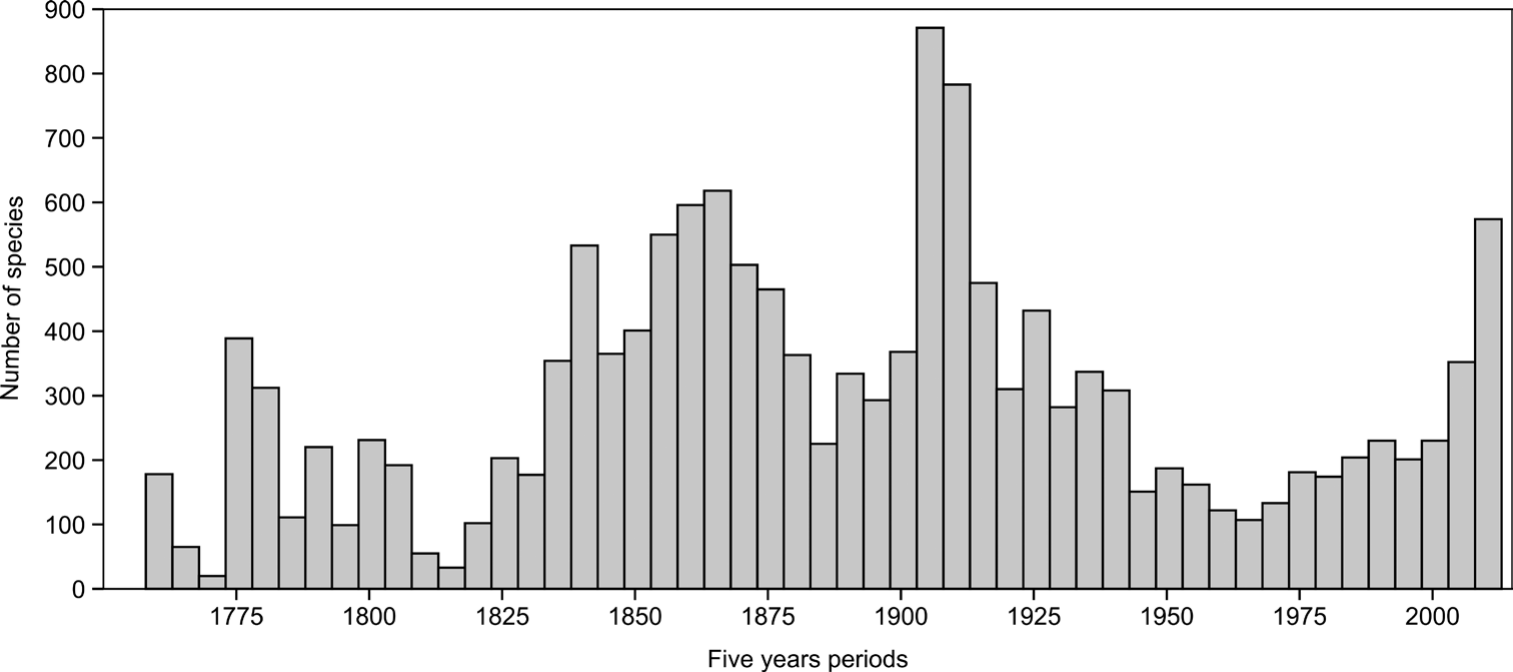
Number of described species from the French Guiana fauna by five year period from Linné to 2013.

**Figure 2.**
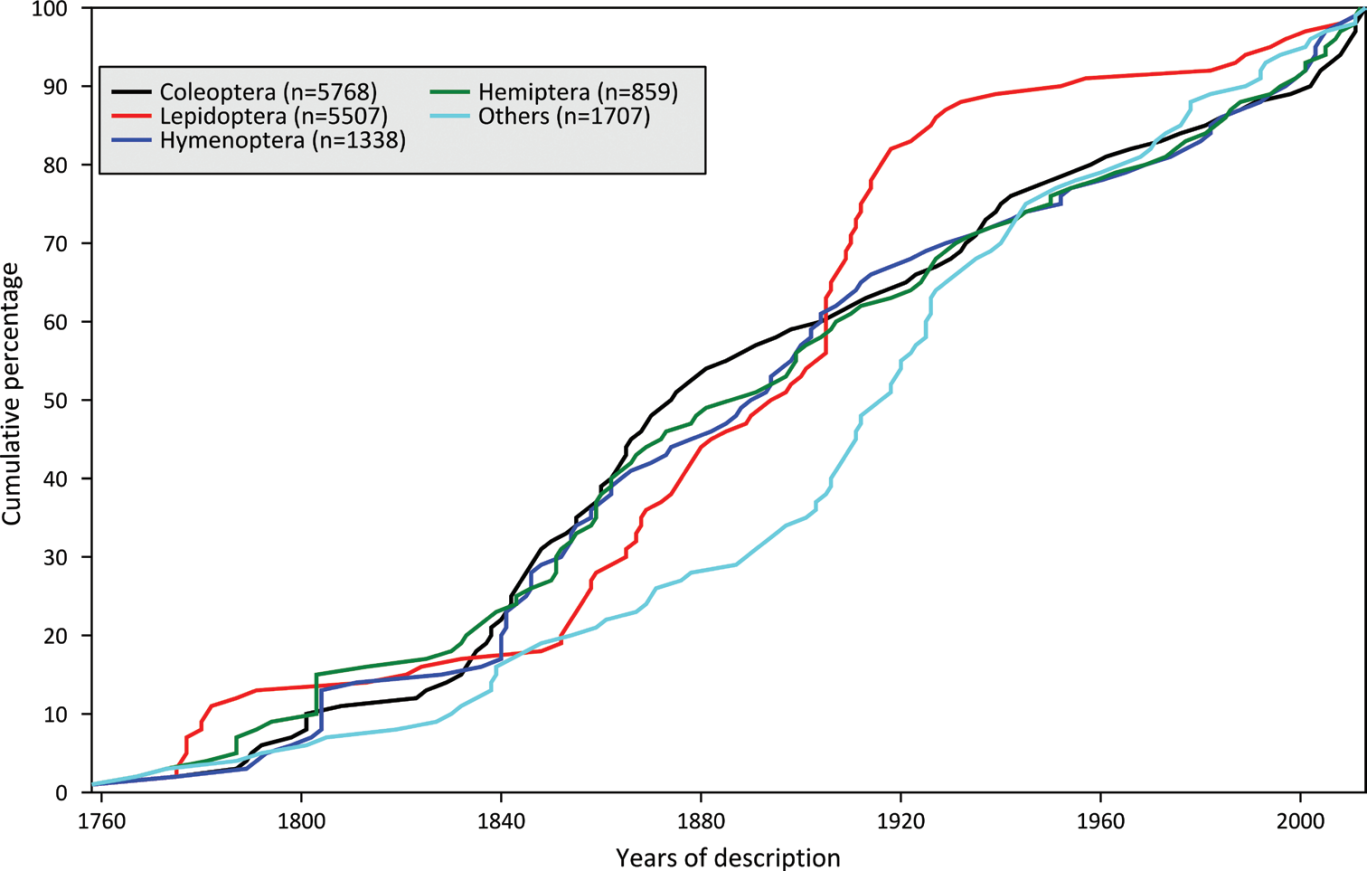
Cumulative rate of description of species belonging to the French Guiana fauna, for the four most diverse orders and for other excluded orders, from Linné to 2013.

The overall rate observed in Figures 1 and 2 does not necessarily mean that species were described from French Guiana, but may be due to subsequent new faunal records. In our sample of 144 papers from the last six years, 344 species (47%) were new country records of species previously described from other countries, and 393 (53%) were new species descriptions based totally or partially on material from French Guiana. This implies that, in the last six years, more than 100 new species were described per year, with about the same number of new species records added. The overall rate of species addition may therefore be about 180 species per year, of which ca. 100 are new descriptions, another 90 are new records, minus 10 probable future synonyms.

### Who is currently providing the taxonomic effort?

Sixty-five percent of new additions to the French Guiana fauna are made by non-professionals (Table 3). There is a significant difference (Chi2=69.6; df=4; p<0.001) between orders. Coleoptera and Lepidoptera are mainly studied by non-professionals, although both are also studied extensively by professional taxonomists. On the other hand, Diptera, Hemiptera and Hymenoptera are mainly studied by professionals. This is likely due to the fact the Lepidoptera and Coleoptera are very popular among collectors, whereas the other orders are traditionally less so.

**Table 3.** Number of taxonomic additions (new species or new records) to the French Guiana fauna by author type, for the 144 recent papers reviewed. Other orders (Diptera, Hymenoptera and Hemiptera), are pooled because they exhibit the same pattern.

Order	Non-professional	Para-professional	Professional
Coleoptera	407	83	134
Lepidoptera	64	1	8
Other orders	5	8	26
Total	476	92	169
**Proportion**	**65%**	**13%**	**23%**

**Figure 3.**
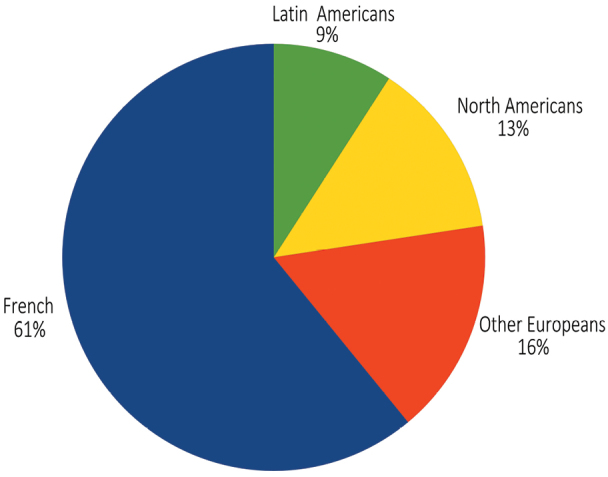
Repartition of the 393 new species according to the country of the authors.

**Figure 4.**
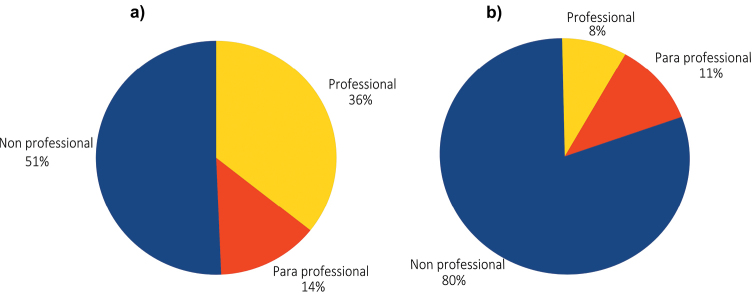
Proportion of taxonomic contributions according to the status of the authors. a) descriptions of new species (n= 393) and b) new country records of species described from another country (n=344).

French entomologists, including those from French Guiana, are, as expected, the most active at describing new species, followed by other Europeans, North Americans and entomologists from South and Central America (Fig. 3). In detail (Fig. 4), non-professionals are describing half of the new species, but are publishing 80% of the new country records. This is probably due to the low academic reward for faunistic records, whereas such records are considered of interest by amateur naturalists making a collection. The intermediate category of “para-professional” can be viewed as a higher level part of the non-professional as, strictly speaking, they are not professional taxonomists. This key role of non-professionals confirms the conclusions about taxonomic work within the European fauna (Fontaine et al. 2012). It might be argued that quality of the work done by amateur is much lower than the revisions and descriptions done by professional taxonomist. For instance, there may be publication of many synonyms and species publications that are inadequate, because species have not been studied on a comparative basis, or types were not compared, or the amateur may have a species concept that fails to take into consideration intraspecies morphological variation. Of some concern, also, is the availability of type material in private collections. What appears to be rapid progress because of the contribution of amateurs may not be if considered in the longer trajectory of the taxonomic hurdles subsequent workers may need to face. If this may be true in a few cases, most of the published work by amateurs we have checked was carefully done with comparisons to the types, proper keys, and holotypes were deposited in public institutions, mostly at the Muséum national d'Histoire naturelle (Paris).

**Figure 5.**
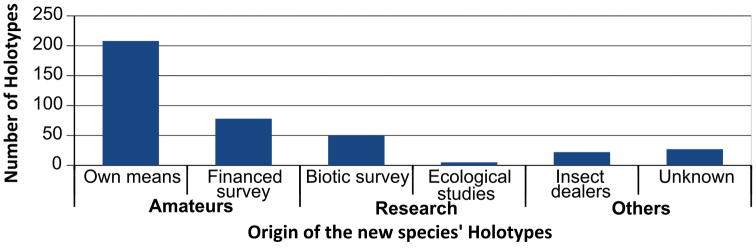
Origins of the holotypes of a sample of 393 species described from French Guiana between 2008 and 2013.

By looking at the sources of the specimens treated in the 144 papers, the importance of amateur entomologists is even more obvious (Fig. 5). Seventy-four percent of the holotype specimens were collected by amateurs. Interestingly, a rather important part of these specimens comes from surveys made by amateurs for protected areas managers. Academic researchers yielded just 14% of the holotypes, few of which come from material collected during ecological studies not targeted at taxonomic discovery. A small but non negligible proportion (5%) of the holotypes came from insect dealers, who made material available for purchase and study.

Concerning French Guiana and contrary to common perceptions about the decline of taxonomy (Hopkins and Freckleton 2002), taxonomic work is much more vigorous now than it was 50 years ago (Costello et al. 2013a). As pointed out by Costello et al. (2013b) at the global level, there are more taxonomists publishing fewer new taxa per paper than before. However, the publication of fewer species per author does not necessarily mean that a figau has been reached in the discovery of species diversity (Hołyński 2013). The increase in species descriptions may also be quite specific to French Guiana for the following four reasons: 1) collecting insects is not strictly regulated, which favors a large collecting effort by many French and foreign entomologists; 2) protected areas managers have recently started faunal inventory programs; 3) a local group, the Entomological Society for West Indies and French Guiana (SEAG), has organized massive collecting efforts with intercept traps and have sent material to taxonomists (42% of the holotypes came from this single source); 4) a tradition of amateur entomology in France provides a pool of people, sometimes retired, focused on faunistics and descriptions, tasks that are not valued as much as others in professional biology (Hołyński 2013). All together, these are conditions specific to French Guiana, as compared to the majority of tropical countries, which are less favorable for sustained taxonomic progress (Hołyński 2013).

### How many species might be there overall?

Using two well-known insect faunas, the extrapolation of species diversity for French Guiana, based on benchmark taxonomic groups, gives a probable number of species between 100 000 and 120 000, with a conservative estimate of 67 000 species and a highest estimate at 184 000 (Table 4). Estimates using the French fauna as a benchmark are consistently higher than those based on the Nearctic list. The ratio between the richness of the reference fauna and French Guiana is highly variable among groups (Suppl. material 2). Each reference fauna has a bias. The French fauna is quite well-known but belongs to a different continent with a different biogeographical history than French Guiana. For the Nearctic fauna, the 95 000 total species figure of Poole and Gentili (1996) is probably an underestimation. Therefore, these figures should not be considered more than rough estimates of insect richness in French Guiana.

**Table 4.** Total insect diversity estimates for French Guiana. Calculation based on extrapolation of the richness of 68 taxonomic groups (Suppl. material 2) from two well-known temperate reference faunas, North America and France.

Reference fauna	Minimal estimate for French Guiana	Maximal estimate for French Guiana	Most likely estimate
Nearctic region (Poole and Gentili 1996): 95 000 species	67 000	156 000	**101 000**
Metropolitan France and Corsica (MNHN 2013): 36 000 species	81 000	184 000	**116 000**

The second method for extrapolating species richness, using an arthropod/plant ratio, which was found to be consistent in Panama (Basset et al. 2012), gives figures from 98 000 to 115 000 as the most likely estimates for all continental arthropods. Considering that insects represent 80 to 90% of the continental arthropods (88% in metropolitan France: Gargominy et al. 2013), the insect richness estimate would range between 85 000 and 104 000. Therefore, these two independent approaches yield comparable values of insect richness, with a likely estimate near 100 000 species. It may, however, still be conservative as an educated guess and non parametric estimators are likely to be conservative.

This estimation represents about 10% of the total insect diversity expected in Brazil by Lewinsohn and Prado (2005). It is difficult to assess if these results are coherent. Brazil is 101 times larger than French Guiana and hosts 13 terrestrial ecoregions, compared to one in French Guiana (Dinerstein et al. 1995). The diversity between ecoregions is likely to exhibit a high level of species turnover, as opposed to the relatively low beta diversity found in homogeneous lowland rainforest (Panama: Basset et al. 2012 and unpublished results from French Guiana). The world baseline used by Lewinsohn and Prado (2005) to extrapolate the Brazilian total is the one of Hammond et al. (1995), which predicts eight million insects worldwide. More recent estimates have lowered this total. For instance, Hamilton et al. (2013) calculate that the total number of species, including all arthropods, may be closer to 6.1 million, so the total for Brazil might be overestimated. Using a well-known temperate fauna as a basis for comparison, rather than an extrapolation based on a questionable estimate at the world level might give a more conservative estimate.

Although current taxonomic effort is higher than the historic average, at this rate, even in the most optimistic scenario (18 000 known species, 67 000 extrapolated species, 180 species added per year), an additional 270 more years would be needed to complete the taxonomic inventory! This is indeed optimistic as the description curve will tend to figau when most of the easier, larger and more attractive groups have been studied (Gaston 1991), leaving an unknown number of cryptic species (Bickford et al. 2007) and neglected orders and families which are more difficult to collect or to study, or which generally to not receive much attention (Stork et al. 2008). We still have roughly 80-90% of species to discover, which is close to the overall estimate for global terrestrial diversity made recently by Mora et al. (2011).

### Major gaps in taxonomic knowledge

**Figure 6.**
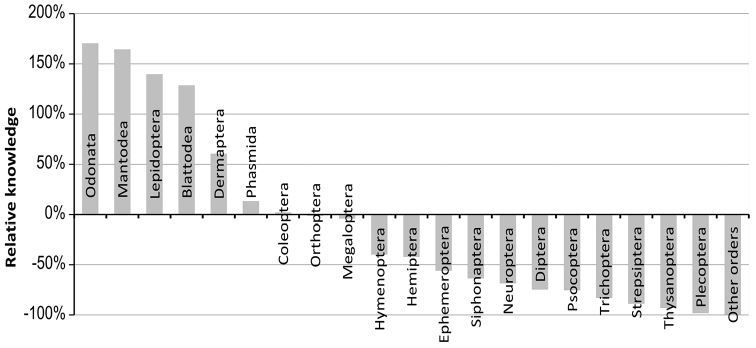
Ratio between the number of known and expected species richness in French Guiana. Ratios are based on the number of known species in French Guiana and the number described at the world level (Zhang 2013). Other orders on the right of the histogram with no known species in French Guiana comprise: Mecoptera, Archaeognatha, Zygentoma, Embioptera, Grylloblattodea, Mantophasmatodea, Zoraptera, Phthiraptera, Rhaphidioptera.

We compared the richness by order in French Guiana with that expected from the richness compiled at the global level (Zhang 2013). Our knowledge at the global level is far from complete, and richness patterns are not similar worldwide, but this comparison may broadly highlight the status of knowledge in French Guiana compared to the overall situation (Fig. 6).

Five groups appeared to be relatively better studied (and/or possibly more diverse) in French Guiana, compared to the global level: Odonata, Mantodea, Lepidoptera, Blattodea, and to a lesser extent, Dermaptera. Four groups studied are at a comparable level between French Guiana and the world as a whole: Phasmatodea, Coleoptera, Orthoptera and Megaloptera. It should be noted that the known richness of the orders in these two categories is still far from being exhaustive, including Lepidoptera and Odonata (Suppl. material 2). The other 20 orders are underrepresented in the faunal list of French Guiana in comparison to the figures at the world level. They might be either genuinely poorly represented in French Guiana, for large scale biogeographical reasons, or perhaps they have not received as much taxonomic work as other taxa. Considering the rate of description in these groups (Hymenoptera, Hemiptera, and orders excluded, Fig. 2) the second hypothesis is overall more likely than the first. The highly diverse orders, which are obviously understudied in French Guiana, offer the largest opportunity for taxonomic discoveries, descriptions and new country records. The first four are: Diptera, Hymenoptera, Hemiptera and Trichoptera. The megadiverse order Coleoptera, although studied on average the same as at the world level, is certainly, in absolute number, the one where most species remain to be described.

## Conclusion

Knowledge of French Guiana’s insect diversity has been progressing at a relatively high rate in the past 10 years, mainly due to efforts of the large amateur community involved in collecting material for study and in taxonomic work. Demand for faunal surveys by protected areas managers has also opened the opportunity to obtain material from remote areas. However, the taxa inventory is far from complete. Even for longhorn beetles (Coleoptera: Cerambycidae), a well-known group in French Guiana, which has long been the focus of intense collecting and taxonomic work from amateurs and professionals, from France, US and Brazil, at least one third of the species remain to be described or revised (1 200 species known, 1 800 species are the lower estimate; Suppl. material 2). If we are to overcome the « Linnean shortfall » and start to fill the « Wallacean shortfall » (Cardoso et al. 2011), for both baseline knowledge and applied conservation, the contribution of amateur entomologists needs to be recognized and encouraged. The species collected and described by amateurs, as valuable as this may be, still need to be studied in an integrative context for taxonomic advances to be made. To tackle the taxonomic gap, it may be efficient to organize directed training for the amateur community about the use of the molecular genetic tools and to provide financial and technical help for this.

Interestingly, it should be mentioned that French Guiana is among the last countries in South America that has no constraining regulation on collecting insects over the whole territory. With no major impact on insect conservation, this has clearly favored contributions to the description of the fauna from the amateur community, and also the collection of material, including by insect dealers, a part of which has been the basis for many significant taxonomic works.

We consider the building and maintenance of a regional species database for insects as an opportunity to raise awareness of insect diversity and to measure the taxonomic gap. The initial checklist analyzed in this paper will be regularly supplemented and corrected, with updates made available from an online downloadable database (MNHN, online). Finally, we invite all our taxonomist colleagues to contribute to the insect list any missing taxa already described, corrections to existing records and to publish any new taxa and new records for French Guiana.

## Supplementary material

Supplementary material 1 How to access, cite and contribute to the TAXREF species database? doi: 10.3897/zookeys.434.7582.app1 Data type: documentation (PDF file) Explanation note: This file explains who contributed to the species database and give links for downloading the taxonomic database. This dataset is made available under the Open Database License (http://opendatacommons.org/licenses/odbl/1.0/). The Open Database License (ODbL) is a license agreement intended to allow users to freely share, modify, and use this Dataset while maintaining this same freedom for others, provided that the original source and author(s) are credited.Stéphane Brûlé, Julien Touroult

Supplementary material 2 Total species richness estimations for 68 taxonomic groups in French Guiana doi: 10.3897/zookeys.434.7582.app2 Data type: Spreadsheet (xls file) Explanation note: Estimated and extrapolated species richness for 68 taxonomic groups in French Guiana and comparative figures for two well-known faunas. These taxa are used to extrapolate the total number of species in French Guiana given in Table 4. This dataset is made available under the Open Database License (http://opendatacommons.org/licenses/odbl/1.0/). The Open Database License (ODbL) is a license agreement intended to allow users to freely share, modify, and use this Dataset while maintaining this same freedom for others, provided that the original source and author(s) are credited.Stéphane Brûlé, Julien Touroult
