# Supplementary material for: Insects of French Guiana: a baseline for diversity and taxonomic effort
Source: Zookeys. 2014 Aug 14;(434):111–30. doi: 10.3897/zookeys.434.7582 (PMC4141168; doi:10.3897/zookeys.434.7582)
Supplement: Supplementary material 1 — How to access, cite and contribute to the TAXREF species database? [file zookeys-434-111-s001.doc]

**Supplementary file 1.**

**How to access, cite and contribute to the TAXREF species database?**

Citation of the electronic checklist for the Insects of French Guiana:

Brûlé S., Aguiar A., Asenjo A., Ballerio A., Barbut J., Barcley M., Batista dos Santos P., Bénéluz F., Bérenger J.-M., Boilly O., Boucher S., Brachat V., Braet Y., Brailovksy H., Cassola F., Chassain J., Cline A., Collet Ph., Constantin R., Dalens P.-H., Degallier N., Delvare G., Deuve T., Durand F., Erwin T., Faynel C., Feer F., Feitosa R., Fernandez S., Foucard A., Gibbs D., Gillung J., Girod C., Gonzales D., Guilbert E., Gustafson G., Guttierez E., Hauser M., Heiss E., Hermier B., Herrmann A., Hevel G., Jameson M. L., Juillerat L., Kneubühler B., Kurina O., Lavalette F., Le Goff G., Leblanc P., Lévêque A., Lohez D., Lupoli R., Mantilleri A., Masner L., Massutti de Almeida L., Morin D., Moron Rios M. A., Olmi M., Paulmier Y., Pape T., Pauly A., Ponchel Y., Queney P., Raper C., Ratcliffe B., Rheinheimer J., Sakakibara A., Soon V., Soulier-Perkins A., Stramare Ribeiro-Costa S., Takyia D., Thomas A., Thouvenot, M., Tomasovic G., Tussac M., Vilhelmsen L., Wachtel F., Wood M., Yvinec J.-H., & Touroult J. 2013. Liste préliminaire des Insectes connus de Guyane. SEAG. In: MNHN (ed.). TAXREF v8.0, référentiel taxonomique pour la France.

<http://inpn.mnhn.fr/programme/referentiel-taxonomique-taxref?lg=en>

Avalaible online after free registration:

<http://inpn.mnhn.fr/programme/referentiel-taxonomique-taxref?lg=en>

**This updated species database with the French Guiana fauna will be available online by september 2014, on the 8^th^ edition of the species database of French species.**

Corrections, modifications, additions are welcome and can be sent to the authors :

Julien Touroult : [touroult@mnhn.fr](mailto:touroult@mnhn.fr)

Stéphane Brûlé : [stephanebrule973@hotmail.fr](mailto:stephanebrule973@hotmail.fr)
